# A narrative review on environmental impacts of cannabis cultivation

**DOI:** 10.1186/s42238-021-00090-0

**Published:** 2021-08-06

**Authors:** Zhonghua Zheng, Kelsey Fiddes, Liangcheng Yang

**Affiliations:** 1 Department of Civil and Environmental Engineering, University of Illinois at Urbana-Champaign , Urbana, IL 61801 USA; 2 Department of Health Sciences Environmental Health and Sustainability Program, Illinois State University, Normal, IL 61790 USA

**Keywords:** Cannabis cultivation, Water demand, BVOCs emission, Carbon footprint, Soil erosion

## Abstract

Interest in growing cannabis for medical and recreational purposes is increasing worldwide. This study reviews the environmental impacts of cannabis cultivation. Results show that both indoor and outdoor cannabis growing is water-intensive. The high water demand leads to water pollution and diversion, which could negatively affect the ecosystem. Studies found out that cannabis plants emit a significant amount of biogenic volatile organic compounds, which could cause indoor air quality issues. Indoor cannabis cultivation is energy-consuming, mainly due to heating, ventilation, air conditioning, and lighting. Energy consumption leads to greenhouse gas emissions. Cannabis cultivation could directly contribute to soil erosion. Meanwhile, cannabis plants have the ability to absorb and store heavy metals. It is envisioned that technologies such as precision irrigation could reduce water use, and application of tools such as life cycle analysis would advance understanding of the environmental impacts of cannabis cultivation.

## Background

The*Cannabis* plant has been cultivated throughout the world since ancient civilizations and used for thousands of years for both medicinal and recreational applications. Cannabis contains a psychoactive compound called tetrahydrocannabinols (THC) that creates a psychogenic effect. It can be consumed through the respiratory tract and digestive tract through smoking and oral ingesting, respectively. In contrast, cannabidiol (CBD), another component derived from cannabis, is a non-psychoactive cannabinoid that has gained popularity for its medicinal values and as a supplement. In the USA, an estimated “30 million Americans use marijuana (cannabis) at least occasionally, and 20 million use it at least once per month” (Osbeck and Bromberg 2017). Despite being used widely, the lack of science-based information due to the legal status of cannabis in the last centuries worldwide (e.g., in the USA) has prevented research.

Cultivation methods have an unavoidable influence on the environment in different degrees. Outdoor cultivation is the traditional and original method of cannabis cultivation. Although with low costs, it is subject to weather and natural resources. Improper soil and water resources management and pest control may induce critical environmental issues. On the contrary, indoor cultivation (including greenhouse cultivation) enables full control over all aspects of the plants, such as light and temperature, but is constrained by higher costs, energy demand, and associated environmental implications. Reducing the global environmental impact of agriculture is vital to maintain environmental sustainability. However, there is a lack of systemic principles towards the sustainable farming of cannabis because its environmental impacts remain unclear. In the wake of the unprecedented legalization of cannabis, there is a pressing need for a complete review of its environmental assessment.

In this paper, we conduct a narrative review of the available literature. We strive to build a better understanding of the environmental impacts induced by cannabis cultivation. This improved understanding can benefit communities, including policymakers, cannabis industry stakeholders, agricultural engineers, ecologists, and environmental scientists. This review covers the environmental effects on water, air, and soil. Energy consumption and carbon footprint are included as well. Possible research directions are also put forward.

## Methods and materials

The literature search for this narrative review paper was conducted several times in 2020 and 2021. We searched combinations of keywords such as “cannabis cultivation,” “marijuana cultivation,” “cannabis water demand,” “cannabis emissions,” “cannabis energy demand”, and “environmental impacts.” Papers, reports, and government documents from 1973 to 2021 from Science Direct and Google Scholar databases have been searched in English. We screened over 250 literatures and discarded irrelevant literature for further analysis. A total of 63 literatures were cited in the review.

### Water demand analysis

To unify the water demand calculations from different data sources, we conducted the following unit conversions:

1 $$1 \mathrm {inch of water}=\mathrm {27,154} \mathrm {gallons of water per acre}$$

2 $$1\mathrm{ acre}=\mathrm{43,560} {\mathrm{ft}}^{2}$$

Similarly, units reported for water demand such as “mm/total growing period” were converted to “gallon/ft^2^/day”. For example, the water need of cotton is 700 mm per total growing period. The water demand was calculated to:

3 $$700 \mathrm{mm} =27.56 \mathrm {inches}=\mathrm {748,346} \mathrm {gallon per acre}$$

Finally, the minimal daily water demand for cotton (shown in Table 1) was calculated using the maximal growing days (195 days):

**Table 1:** Water demand comparison between Cannabis and commodity crops

Plants	Total growing period (*days*)	Water demand per season (*million gallons acre*^*−1*^)	Daily water demand (*gallon ft*^*−2*^* day*^*−1*^)	Ref
Cannabis: outdoor	150	1.57 ^a^	0.24	(HGA, 2010)
Cannabis: outdoor	August	n.a	0.22	(Wilson et al., 2019)
Cannabis: outdoor	September	n.a	0.17	(Wilson et al., 2019)
Cannabis: indoor	August	n.a	0.18	(Wilson et al., 2019)
Cannabis: indoor	September	n.a	0.22	(Wilson et al., 2019)
Cotton	180–195	0.75–1.39^b^	0.09–0.15	(Brouwer and Heibloem, 1986)
Cotton	/	/	0.14–0.17	(Hussain et al., 2020)
Maize	130–150	0.53–0.86^b^	0.07–0.13	(Brouwer and Heibloem, 1986)
Corn	/	/	0.22 (peak)	(Rogers et al. 2017)
Soybean	135–150	0.48–0.75^b^	0.07–0.13	(Brouwer and Heibloem, 1986)
Soybean	/	/	0.22 (peak)	(Rogers et al. 2017)
Wheat	120–150	0.48–0.69^b^	0.07–0.19	(Brouwer and Heibloem, 1986)
Wheat	/	/	0.19 (peak)	(Rogers et al. 2017)
Rice	90–150	0.48–0.75^b^	0.09–0.18	(Brouwer and Heibloem, 1986)
Rice	/	/	0.11–0.15	(Intaboot, 2017)

*Note*^a^: The water demand of cannabis is calculated based on 22.7 l (6 gallons) of water per day during the growing season and 200 plants per 5,000 sq. ft (HGA, 2010)

*Note*^b^: The water demand of crops is based on crop water need from Table 14 in Brouwer Heibloem (Brouwer and Heibloem, 1986). We convert the unit from mm to million gallon acre^−1^ according to the rule of unit conversion where 1 acre inch is equivalent to 27,154.29 gallon

4 $$\frac{\mathrm{748,346} \mathrm{gallon per acre}}{195\mathrm{ days}}\times \frac{\mathrm{acre}}{\mathrm{43,560} {\mathrm{ft}}^{2}}=0.09\frac{\mathrm{gallons}}{{\mathrm{ft}}^{2}\times \mathrm{days}}$$

### Water demand and pollution

#### Water demand

Cannabis is a water- and nutrient-intensive crop (Carah et al. 2015). Table 1 shows that the water demand for cannabis growing far exceeds the water needs of many commodity crops. For example, cannabis in a growing season needs twice as much as the water required by maize, soybean, and wheat. On average, a cannabis plant is estimated to consume 22.7 l (6 gallons) of water per day during the growing season, which typically ranges from June to October for an approximate total of 150 days (Butsic and Brenner 2016). As a comparison, the mean water usage for the wine grapes, the other major irrigated crop in the same region, was estimated as 12.64 l of water per day (Bauer et al. 2015). Although the average daily water use varies from site to site, depending on many factors such as the geographic characters, soil properties, weather, and cultivation types, it is an agreed-upon truth that cannabis is a high-use water plant. A survey conducted by Wilson et al. (2019) reports the water usage of outdoor cannabis cultivation in California is 5.5 gallons per day per plant (equivalent to 0.22 gallon ft^−2^ day^−1^) in August and 5.1 gallons per day per plant (equivalent to 0.17 gallon ft^−2^ day^−1^) in September (Wilson et al. 2019). The indoor cultivation water consumptions are 2.5 and 2.8 gallons per day per plant in August and September. However, the application rates (0.18 gallon ft^−2^ day^−1^ in August and 0.22 gallon ft^−2^ day^−1^ in September) are very close to outdoor cultivation (Wilson et al. 2019). In California, irrigated agriculture is regarded as the single largest water consumer, accounting for 70–80% of stored surface water and pumping vast volumes of groundwater (Moyle 2002; Bauer et al. 2015). The great water demand induced by agriculture, amid population growth and climate change, is most likely to exacerbate water scarcity in the foreseeable future (Bauer et al. 2015). Notably, the predicted decrease in water availability downscales in California may adversely affect the value of farmland (Schlenker et al. 2007) and pose a severe challenge to the cannabis industry. As a result, the immense amount of water necessary to keep cannabis plants alive and healthy will continue to burden our environment.

The high water demand presses the need for water sources. Water diversion is a common practice, which removes or transfers the water from one watershed to another to meet irrigation requirements. While the water diversion alleviates the water shortage problem for cannabis cultivation, it also presents new challenges. A study conducted by Bauer et al. quantitatively revealed that surface water diversions for irrigation led to reduced flows and dewatered streams (Bauer et al. 2015). Four northwestern California watersheds were investigated in this study since they are remote, primarily forested, sparsely populated. The results show that the annual seven-day low flow was reduced by up to 23% in the least impacted watersheds of this study, and water demands for cannabis cultivation in three watersheds exceed streamflow during the low-flow period. More recently, Dillis et al. identified well water (58.2%), surface water diversions (21.6%), and spring diversions (16.2%), are the most commonly extracted water source for cannabis cultivation in the North Coast region of California (Dillis et al. 2019). The distributing percentages, however, vary among the counties. For example, the growers in Humboldt County relied more on surface water and spring diversions (57%) than the wells (40.9%), while another study conducted by Wilson et al. showed that groundwater (wells or springs) was the primary water source for irrigation, followed by municipal water, rainwater, and surface water (Wilson et al. 2019).

#### Water pollution

Cannabis cultivation, especially illegal cultivation, may deteriorate water quality. Recent studies have suggested the considerable demands of nutrition such as nitrogen (Saloner and Bernstein 2020, 2021), phosphorous (Shiponi and Bernstein 2021), and potassium (Saloner et al. 2019) for cannabis growth. However, there is limited data on the impact of cannabis cultivation on water quality worldwide or even nationwide. Here we focus on a survey conducted by Wilson et al. (2019) for CA, USA. Based on the survey, more than 30 different soil amendments and foliar nutrient sprays were used to maintain nutrition and fertility (Wilson et al. 2019). The applied pesticides (including herbicides, insecticides, fungicides, nematodes, and rodenticides), due to routine pest and disease controls, make their way into the water without restriction and therefore posing significant risks to the water environment (Gabriel et al. 2013). The transport and fate of the applied fertilizers and pesticides vary. For example, nitrogen and pesticides can get into runoff or leach into groundwater due to rainfall or excessive irrigation (Trautmann et al. 2012). If the polluted water continues to be used, it would add contaminants into soil, surface water, and groundwater. These chemicals may threaten humans and crops through the food chain (Pimentel and Edwards 1982). The other major irrigated crops can also be significantly impacted since the placement of crops is subject to the environmental safety of runoff, groundwater contamination, and the poisoning of nearby bodies of water. However, without the ability to sample water quality and assess the extent to which chemical inputs are entering adjacent water bodies, the ability to link cultivation practices to water pollution is greatly limited (Gianotti et al. 2017). Besides, few environmental clean-up and remediation efforts in the polluted watersheds are accessible due to a lack of resources and staff in state or federal agencies.

#### Water ecosystem

Water diversion and water pollution affect the water ecosystem. The high demand for water due to cannabis cultivation in watersheds affects wildlife such as fish and amphibians in a significant way since cannabis cultivation is widespread within the boundaries of the watersheds, where the downstream water houses populations of sensitive aquatic species. The diminished flows may be notably detrimental to salmonid fishes since they need clean, cold water and suitable flow regimes (Bauer et al. 2015). As the reduced streamflow has a strong positive correlation with increased water temperature, indirectly resulting in reduced growth rates in salmonids, lowered dissolved oxygen, increased predation risk, and increased susceptibility to disease (Marine and Cech 2004). It has been reported that there are 80%–116% increases in cannabis cultivation sites near high-quality habitats for threatened and endangered salmonid fish species (Butsic et al. 2018). Besides, the threat of water diversions and altered stream flows to amphibians cannot be neglected. The desiccation-intolerant species, such as southern torrent salamander (*Rhyacotriton variegatus*) and coastal tailed frog (*Ascaphus truei*), are vulnerable to headwater stream diversions or dewatering (Bauer et al. 2015). The headwater stream-dwelling amphibians also exhibit high sensitivity to water temperature changes (Bury 2008). It is vital to get all the growers on the same page regarding water resources because flow modification is one of the greatest threats to aquatic biodiversity. The cannabis industry is becoming a major abuser concerning water diversions. Studies show that the second-generation anticoagulant rodenticides (ARs) affect many predators in both rural and urban settings (Gabriel et al. 2013, 2012; Elliott et al. 2014). Necropsy revealed that a male fisher had died of acute AR poisoning in April 2009, most likely due to the source of numerous illegal cannabis cultivation sites currently found on public lands throughout the western USA (Thompson et al. 2014). A study examining the effects of Ars on the Pacific fisher reports that four out of fifty-eight deceased fishers examined were killed by “lethal toxicosis, indicated by AR exposure.”

### Outdoor and indoor air quality

#### Outdoor air quality

Little attention has been devoted so far to study the impact of cannabis cultivation on outdoor air quality. The emission of volatile organic compounds (VOCs) attracts special attention because of the vital role played by VOCs in ozone and particulate matter formation, as well as VOC’s health impact (D.R. et al. 2001; Jacob 1999). Amongst the VOCs, the biogenic volatile organic compounds (BVOCs) (Atkinson and Arey 2003), mainly emitted from vegetation, account for approximately 89% of the total atmospheric VOCs (Goldstein and Galbally 2007). Previous studies have identified cannabis plant tissues contain high concentrations of many BVOCs such as monoterpenes (C_6_H_16_), terpenoid compounds (e.g., eucalyptol; C_10_H_18_O), sesquiterpenes (C_15_H_24_), and methanol. Hood et al. investigated that the monoterpenes α-pinene, β-pinene, β-myrcene, and d-limonene accounted for over 85% of the detected VOCs emitted, with acetone and methanol contributing a further 10% (Hood et al. 1973; Rice and Koziel 2015; Ross and ElSohly 1996). However, limited systematic studies characterized and accurately quantified volatile emissions during the growing and budding process (Wang et al. 2019b).

To determine the BVOCs emission rates, Wang et al. employed an enclosure chamber and live Cannabis spp. plants during a 90-day growing period considering four different strains of Cannabis spp. including Critical Mass, Lemon Wheel, Elephant Purple, and Rockstar Kush (Wang et al. 2019b). They found the percentages of individual BVOCs emissions were dominated by β-myrcene (18–60%), eucalyptol (17–38%), and d-limonene (3–10%) for all strains during peak growth (Table 2). The terpene emission capacity was determined, ranging from 4.9 to 8.7 μg-C per g dry biomass per hour. The estimation with μg-C per g dry biomass per hour for Denver would result in more than double the existing rate of BVOCs emissions to 520 metric ton year^−1^, leading to 2100 metric ton year^−1^ of ozone, and 131 metric ton year^−1^ of PM (particular matter). However, a high emission can be expected since the better growing conditions contribute to rapid growth and higher biomass yields.

**Table 2:** Composition of BVOCs

BVOCs	30-day (%)	46-day (%)
β-myrcene	26.6–42.6	18.3–59.4
Eucalyptol	18.5–32.8	16.8–37.6
d-limonene	4.4–17.2	3.0–10.0
p-cymene	2.3–12.8	0.6–4.6
γ-terpinene	2.0–9.7	2.8–14.0
β-pinene	0.4–6.9	1.3–3.5
(Z)-β-ocimene	1.3–5.9	0.0
Sabinene	0.0–5.0	0.2–10.9
Camphene	0.0–4.4	0.0–1.0
α-pinene	0.8–4.3	2.7–3.6
Thujene	0.9–3.1	1.2–3.4
α-terpinene	0.0–2.0	0.5–5.4

Note: *BVOCs* biogenic volatile organic compounds

Data adapted from Wang, C. T., Wiedinmyer, C., Ashworth, K., Harley, P. C., Ortega, J., Vizuete, W. (2019b). Leaf enclosure measurements for determining volatile organic compound emission capacity from Cannabis spp. Atmos. Environ., 199, 80–87. (Wang et al., 2019b)

A recent study conducted by Wang et al. was the first attempt at developing an emission inventory for cannabis (Wang et al., 2019a). This study compiled a bottom-up emission inventory of BVOCs from cannabis cultivation facilities (CCFs) in Colorado using the best available information. Scenarios analysis shows that the highest emissions of terpenes occur in Denver County, with rates ranging from 36 to 362 t year^−1^, contributing to more than half of the emissions across Colorado. With the emission inventory, the air quality simulations using the Comprehensive Air Quality Model with extensions (CAMx) show that increments in terpene concentrations could results in an increase of up to 0.34 ppb in hourly ozone concentrations during the morning and 0.67 ppb at night. Given that Denver county is currently classified as “moderate” non-attainment of the ozone standard (USEPA 2020), the air quality control of the CCF operation is essential.

In addition to BVOC emissions, like every crop cultivation in water-sensitive zones, the fertilization of cannabis causes deterioration in air quality. As fertilization is one of the most critical factors for cannabis cultivation, the introduction of excessive nitrogen into the environment without regulation can lead to adverse multi-scale impacts (Balasubramanian et al. 2017; Galloway et al. 2003). Ammonia in the chemical nitrogen fertilizer volatilized from cropland to the atmosphere forms PM via the reaction with acidic compounds in the atmosphere. Besides, the wet and dry deposition of reactive nitrogen consisting of ammonia continuously deteriorates the ecological environment. Both soil acidification and water eutrophication risks could significantly increase because of the nitrogen cascade (Galloway et al. 2003; Galloway et al. 2008).

#### Indoor air quality

Although cannabis can be grown outdoors in many regions of the world, sizeable commercial cultivation can also occur indoors or in greenhouses. Ambient measurements collected inside growing operations pre-legalization have found concentrations as high as 50–100 ppbv of terpenes including α-pinene, β-pinene, β-myrcene, and d-limonene for fewer than 100 plants in the cannabis cultivation facility (Martyny et al. 2013; Atkinson and Arey 2003; Wang et al. 2019a). The study conducted by Spokane Regional Clean Air Agency (SRCAA) measured indoor VOCs in seven flowering rooms and two dry bud rooms across four different CCFs, reporting the average terpene concentration was 361 ppb (27–1676 ppb) (Southwellb et al. 2017).

Samburova et al. analyzed the BVOCs emissions from four indoor-growing Cannabis facilities in California and Nevada (Samburova et al. 2019). They reported the indoor concentrations of measured BVOCs could vary among the facilities, ranging from 112 μg m^−3^ to 5502 μg m^−3^ (Table 3), for a total measured BVOCs of 744 mg day^−1^ plant^−1^. The BVOCs characterization partially agrees with the measurements shown by Wang et al. where β-myrcene is one of the dominated BVOCs emitted by Cannabis, but eucalyptol was not a dominating terpene in this study (Wang et al. 2019b). The obtained emission rates ranged between 0 to 518.25 mg day^−1^ plant^−1^. The largest emission contributors were β-pinene (518.25 mg day^−1^ plant^−1^, 70% of the total BVOCs) α-pinene (142.92 mg day^−1^ plant^−1^, 19% of the total BVOCs), and D-limonene (30.86 mg day^−1^ plant^−1^, 4% of the total BVOCs). Silvey (2019) characterized the overall VOC total terpene mass concentration using sorbent tube sampling and found a higher range between 1.5 mg m^−3^ (office) to 34 mg m^−3^ (trimming room) (Silvey 2019).

**Table 3:** Indoor BVOCs concentrations

BVOCs	Sites	Unit in *ppbv*	Unit in *ug m*^*−3*^	Ref
α-pinene, β-myrcene, β-pinene, and limonene	Growing room	50–100	n.a	(Martyny et al., 2013; Wang et al., 2019a)
Terpenes	Flowering room	30–1600	n.a	(Southwellb et al., 2017; Wang et al., 2019a)
Total BVOCs	Growing room	n.a	112–5502	(Samburova et al., 2019)
Total BVOCs	Curing room	n.a	863–1055	(Cuypers et al., 2017)
Total BVOCs	Purging room	n.a	1005	(Trautmann et al., 2012)

*BVOCs* Biogenic volatile organic compounds

The indoor cannabis (marijuana) grows operations (known as “IMGO”) also pose a risk of potential health hazards such as mold exposure, pesticide, and chemical exposure (Martyny et al. 2013). For example, cannabis cultivations typically require a temperature between 21 and 32 °C, with a relative humidity between 50 and 70% (Koch et al. 2010), while the ventilation rate is often suppressed to limit odor emanating, especially for the illegal cultivation. John and Miller suggested that the houses built after 1980 in Canada are at high risk of moisture-related damage if used as IMGO, and increased moisture levels of the IMGO are associated with elevated mold spore levels (Johnson and Miller 2012). The reports by IOM (IOM 2004) and WHO (World Health Organization) showed that the presence of mold in damp indoor environments is correlated with upper respiratory tract symptoms, respiratory infections, wheeze, cough, current asthma, asthma symptoms in sensitized individuals, hypersensitivity pneumonitis, and dyspnea (WHO 2009). Cuypers et al. conducted a study in Europe, showing that pesticide use in Belgian indoor cannabis cultivation is a common practice, putting both the growers and intervention staff at considerable risk (Cuypers et al. 2017). They found 19 pesticides in 64.3% of 72 cannabis plant samples and 65.2% of 46 carbon filter cloth samples, including o-phenylphenol, bifenazate, and cypermethrin.

### Energy demands and carbon footprint

#### Indoor cultivation energy demands and impacts

As one of the most energy-intensive industries in the USA (Warren 2015), cannabis cultivation results in up to $6B in energy costs annually, accounting for at least 1% of the nation’s electricity (Mills 2012). The cannabis electricity consumption increases to 3% in California (Warren 2015). In Denver, the average electricity use from cannabis cultivation and associated infused product manufacturing increased by 36% annually between 2012 and 2016 (DPHE 2018). As cannabis becomes legalized throughout the country, energy consumption will continue to grow in the foreseeable future.

**Fig. 1:**
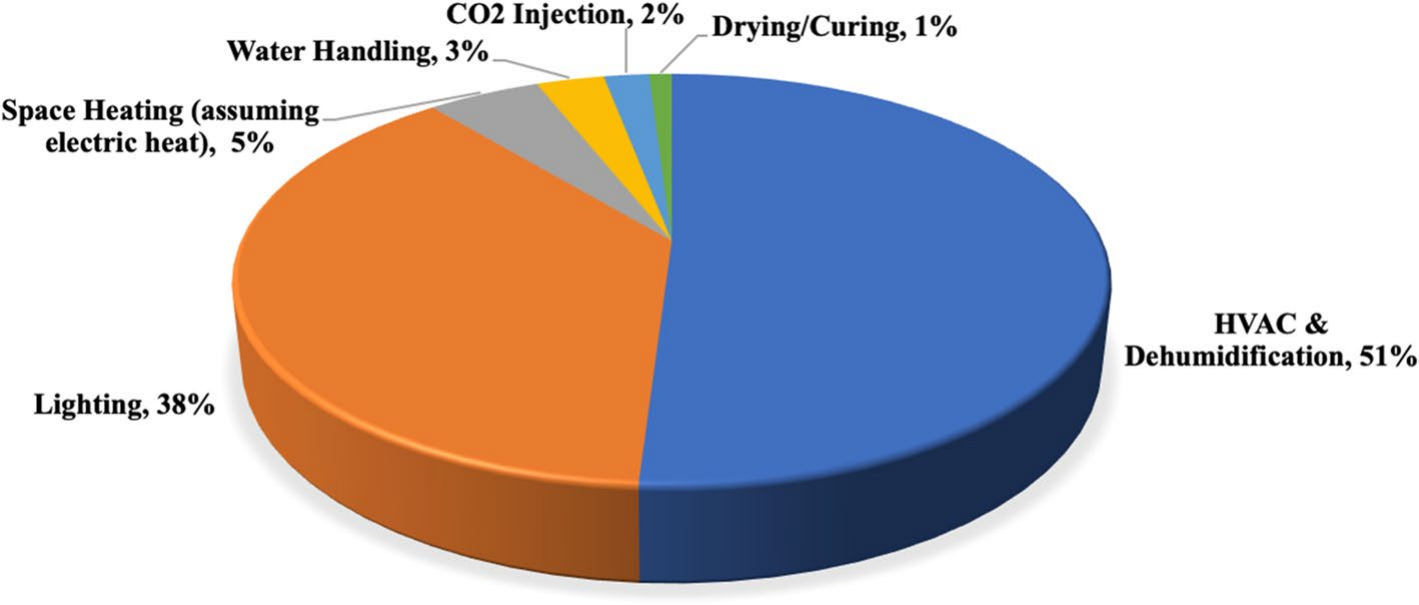
End-use electricity consumption

The energy use of indoor cannabis cultivation arises from a range of equipment, falling into two major categories: lighting and precise microclimate control. For the cannabis plants to thrive and therefore make the growers a profit, several energy-intensive tools are regularly utilized. The energy demand for indoor cannabis cultivation was reported to be 6074 kWh kg-yield^−1^ (Mills 2012). Figure 1 shows the end-use electricity consumption according to a study performed by the Northwest Power and Conservation Council (NPCC 2014). Amongst them, lighting, HVAC (heating, ventilation, and air conditioning), and dehumidification account for 89% of the total end-use electricity consumption.

High-intensity lighting is the main contributor to electricity for indoor production facilities. Sweet pointed out that lighting alone can account for up to 86% of the total electricity usage (Sweet 2016). It has been reported that the intensity of the indoor cannabis lamps (25 klux for leaf phase, and 100 klux for flowering (Mills 2012)) approximates that of hospital operating room lamps, which is up to 500 times greater than a standard reading light (Warren 2015). Indoor cultivation facilities typically utilize a combination of high-pressure sodium (HPS), ceramic metal halide (CMH), fluorescent, and/or light-emitting diode (LED) lamps. In addition to the lamp type, lighting system design is also critical to maximizing energy efficiency in the cultivation facilities, and time of use also plays a crucial role.

HVAC Dehumidification system ensures frequent air exchanges, ventilation, temperature, and humidity control day and night. This system can account for more than half of the total energy consumption in an indoor cultivation facility (Mills 2012). Besides, water and energy are inextricably linked, given water and wastewater utilities contribute to 5% of overall USA electricity consumption (Pimentel and Edwards 1982). The grow systems (including automation and sensors), irrigation (including fertigation and pumps), and CO_2_ injection also consume an amount of electricity.

Energy production, especially fossil fuel use, is accountable for the environmental impact. Table 4 shows that coal and natural gas make up almost three-quarters of the power supply for Colorado customers in the USA. Considering the environmental impacts of different energy sources, the extensive usages of fossil fuels (coal, natural gas, and oil) causes serious environmental damage and pose effects on (1) humans, (2) animals, (3) farm produce, plants, and forests, (4) aquatic ecosystems, and (5) buildings and structures (Barbir et al. 1990).

**Table 4:** Power supply mix for Colorado customers

Energy sources	Total generation mix (%)
Coal	44
Natural gas	28
Wind	23
Solar	3
Hydroelectric	2
Others (including biomass, oil and nuclear generation)	0

Data adapted from Dever Publich Health Environment. 2018. Cannabis Environmental Best Management Practices Guide. (DPHE, 2018)

#### Carbon footprint

The term *carbon footprint* refers to “a measure of the exclusive total amount of carbon dioxide emissions that is directly and indirectly caused by an activity or is accumulated over the life stages of a product” (Wiedmann and Minx 2008). In the context of cannabis cultivation, a carbon footprint can be defined as the total amount of greenhouse gases (GHGs) emitted during the production of cannabis. Denver Department of Public Health Environment broke the GHG inventory down into the three primary scopes: (1) an organization’s direct GHG emissions produced on-site; (2) an organization’s off-site carbon emissions, or indirect emissions; (3) all other indirect carbon emissions associated with the operation of a business (DPHE 2018). However, a relatively small body of literature pays particular attention to the carbon footprint calculation. Mills estimates that producing one kilogram of processed cannabis indoors leads to 4600 kg of CO_2_ emissions to the atmosphere, equivalent to one passenger vehicle driven for one year or 11,414 miles driven by an average passenger vehicle (Mills 2012). Amongst them, the emissions factor (kg CO_2_ emissions per kg yield) of lighting is 1520 (33%), followed by ventilation and dehumidify (1231, 27%), and air conditioning (855, 19%). On the other hand, outdoor cultivation can alleviate the energy use for lighting and precise microclimate control but requires other facilities and techniques such as water pumping. Carbon footprint analysis is the first step towards the carbon reduction strategies, which contributes to the reduction of the environmental impacts of the cannabis industry. Future studies are foreseen to improve the understanding of the carbon footprint of cannabis cultivation both indoors and outdoors.

### Soil erosion and pollution

#### Soil erosion

Soil erosion is a natural process that occurs when there is a loss or removal of the top layer of soil due to rain, wind, deforestation, or any other human activities. It increases fine-sediment loading into streams and threatens rare and endangered species (Carah et al. 2015). Soil erosion can happen slowly due to wind or quickly due to the heavy rainfall event. Land terracing, road construction, and forest clearing make their ways to remove native vegetation and to induce soil erosion (Carah et al. 2015). Barringer (Barringer 2013) and O’Hare et al. suggested that cannabis cultivation directly contributes to soil erosion (O'Hare et al. 2013). The slope is a useful proxy for erosion potential since soil on steep slopes tends to erosion when cleared or cultivated (Butsic et al. 2018). Butsic and Brenner conducted a systematic, spatially explicit survey for the Humboldt County, California, involving digitizing 4,428 grow sites in 60 watersheds (Butsic and Brenner 2016). About 22% of the clustered cannabis on steep slopes indicates a risk of erosion. Many studies also suggest that cannabis cultivation can result in deforestation and forest fragmentation (Wang et al. 2017), which exacerbate soil erosion. Though greenhouse prevents soil erosion, they are surrounded by large clearings accumulated during construction with exposed soils subject to erosion (Bauer et al. 2015).

#### Phytoremediation potential

Cannabis has gradually garnered attention as a “bioremediation crop” because of its strong ability to absorbing and storing heavy metals (McPartland and McKernan 2017). It can remove heavy metal substances from substrate soils and keep these in its tissues by means of its bio-accumulative capacity (Dryburgh et al. 2018). Usually, it takes up high levels of heavy metals from the soil or growing medium via its roots and potentially deposits into its flowers (Seltenrich 2019). Tainted fertilizer uptake from the soil is often a source of heavy metals contamination such as arsenic, cadmium, lead, and mercury. Singani and Ahmadi reported that *Cannabis sativa* could absorb lead and cadmium from soils amended with contaminated cow and poultry manures (Singani and Ahmadi 2012). Though limited studies discussed the effectiveness of cannabis for heavy metals removal, many studies have addressed the uptake of heavy metals by industrial hemp (Campbell et al. 2002; Linger et al. 2002). It indicates that the cannabis plant is qualified as a phytoremediation of contaminated soils.

### Conclusions and envisions

**Fig. 2:**
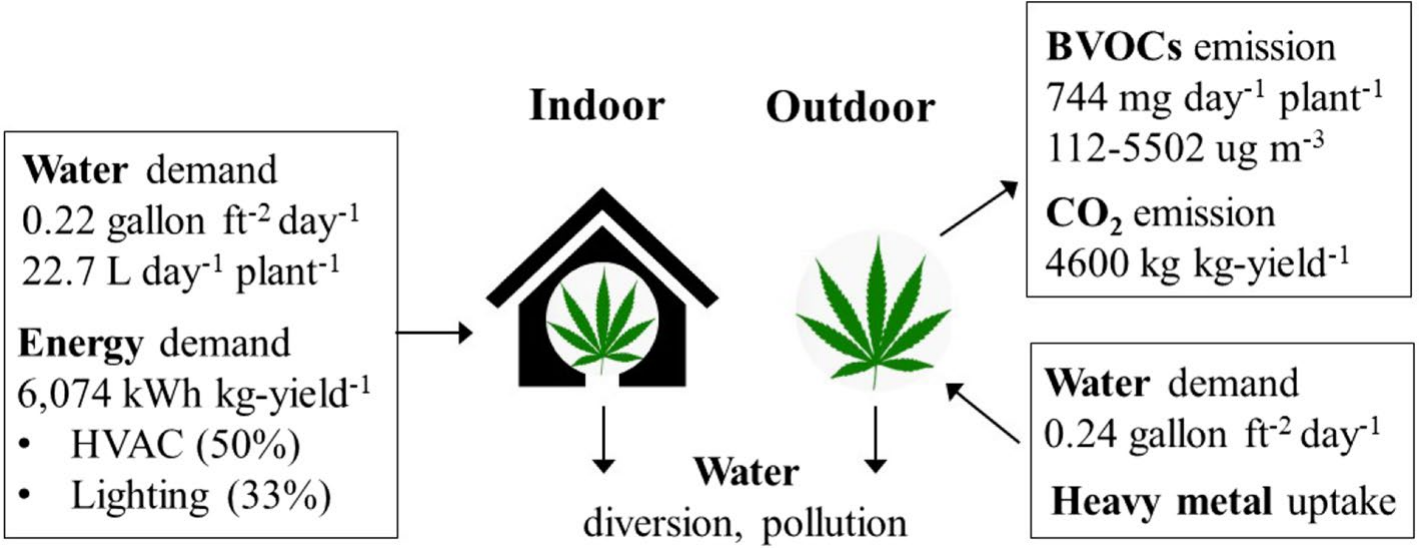
Summary of cannabis environmental impacts

A summary of the environmental impacts of cannabis cultivation is shown in Fig. 2. Water demand and usage will continue to be a major concern. Illegal cannabis cultivation and improper operation may raise water pollution issues. Studies on cannabis’ physiological properties will guide to determine water demand. Besides, identifying and applying best management practices, such as precision irrigation and enhanced climate control, will be critical to minimize the environmental impacts on water. Energy consumptions mainly come from the equipment operation of the indoor cultivations such as lighting, HVAC, and dehumidification. Carbon footprint can be calculated both indoors and outdoors based on energy consumption. Quantitatively accounting for the energy assumption across operations at scales is the key to better estimating the carbon footprint. Techniques such as life cycle energy assessment and life cycle carbon emissions assessment would offer informative guidance to reduce the environmental impacts. Few studies have focused on the impacts of cannabis cultivation on air quality. Evidence has emerged that BVOCs and fertilization may contribute to outdoor air quality issues. Indoor air pollutants, i.e., BVOCs emission, mold, pesticide, and chemicals pose a risk of health hazards. Field or chamber studies on determining the species and emission rate of BVOCs, trace gases, and particles from the plant, plant detritus, and soils are important. Much work will be needed to include this information in the emission inventory for air quality modeling. Investigation concerning the contribution of those species to regional, even global air quality, is useful for policymakers and the public. Besides, a better understanding of indoor pollutant concentration and emission ensures the safety of indoor operation. The environmental impact of cannabis cultivation on soil quality has two sides, and it needs to be treated dialectically. On one side, cannabis cultivation directly contributes to soil erosion. On the other side, cannabis has a strong ability to absorb and store heavy metals in the soil. Further studies on the soil mechanics and dynamics of heavy metals in plant-soil interactions are needed.

## Data Availability

All data generated or analyzed during this study are included in this published article.

## Abbreviations

ARs: Anticoagulant rodenticides
BVOCs: Biogenic volatile organic compounds
CAMx: Comprehensive Air Quality Model with extensions
CBD: Cannabidiol
CCFs: Cannabis cultivation dacility
CMH: Ceramic metal halide
CSA: Controlled Substances Act
GHGs: Greenhouse gases
HPS: High-pressure sodium
HVAC: Heating, ventilation, and air conditioning
IMGO: Indoor Marijuana Grows Operations
LED: Light-emitting diode
NIH: National Institutes of Health
OSHA: Occupational Safety and Health Administration
PM: Particular matter
SRCAA: Spokane Regional Clean Air Agency
THC: Tetrahydrocannabinols
USDA: Department of Agriculture
VOCs: Volatile organic compounds
WHO: World Health Organization
